# Nutritional indicator-based prediction model for esophagogastric variceal bleeding in cirrhosis using XGBoost and SHAP

**DOI:** 10.3389/fnut.2026.1911832

**Published:** 2026-10-06

**Authors:** Dan Zhao, Zhifang Fan, Yujie Yan, Qiaoli Guo, Qianqian Xu, Zhifen Hao, Huifang Huang

**Affiliations:** 1 Department of Gastroenterology, First Hospital of Shanxi Medical University, Taiyuan, China; 2 Department of Clinical Nutrition, First Hospital of Shanxi Medical University, Taiyuan, China

**Keywords:** bleeding, cirrhosis, esophagogastric varices, nutritional screening tools, predictive model, SHAP interpretation, XGBoost

## Abstract

**Purpose:**

Malnutrition is common in cirrhotic patients and associated with poor prognosis, yet current models have suboptimal predictive performance for esophagogastric variceal bleeding (EGVB). This study aimed to construct a noninvasive prediction model integrating nutritional indicators.

**Methods:**

We retrospectively analyzed 416 cirrhotic patients (135 with EGVB, 281 without EGVB) at the First Hospital of Shanxi Medical University between March 2023 and March 2025. Eight nutritional screening tools were compared by univariate logistic regression and receiver operating characteristic (ROC) analysis to identify the optimal tool. An XGBoost prediction model was then constructed with permutation-importance-based feature selection. Model performance was assessed by AUC, accuracy, sensitivity, specificity, and F1-score, with SHAP for interpretation.

**Results:**

Among the eight nutritional assessment tools, univariate logistic regression analysis revealed that MUST (OR = 1.44, 95%CI: 1.22–1.71, *p* < 0.001) achieved the highest predictive accuracy (AUC = 0.698). The optimal XGBoost model incorporated hemoglobin, MUST score, AST, serum creatinine, adjusted BMI, ALT, neutrophils, potassium (K^+^), monocyte count and mid-arm muscle circumference (MAMC). The model yielded an AUC of 0.860, accuracy of 84.3%, recall of 66.7%, precision of 81.8%, F1-score of 80.9%, and Brier score of 0.122. SHAP analysis demonstrated that three nutritional indicators, namely MUST score, adjusted BMI and mid-arm muscle circumference, were important predictors of EGVB. A higher MUST score, higher adjusted BMI, and lower mid-arm muscle circumference were associated with an increased risk of EGVB.

**Conclusion:**

The XGBoost-SHAP model integrating nutritional indicators accurately predicts EGVB risk in cirrhosis, outperforming single traditional nutritional screening tools. This explainable, noninvasive machine learning approach provides a reliable tool for risk stratification and individualized nutritional intervention, potentially improving outcomes in this high-risk population.

## 1 Introduction

Cirrhosis represents the terminal stage of chronic liver injury resulting from various etiologies. Its clinical manifestations primarily include ascites, gastric varices (GOV) with rupture bleeding (esophagogastric variceal bleeding, EGVB), and hepatic encephalopathy (HE). Among these complications, EGVB carries the highest mortality rate and constitutes one of the most common gastrointestinal emergencies (1). Current research indicates that malnutrition affects approximately 20–50% of cirrhotic outpatients and 40–90% of hospitalized patients (2, 3). The American Association for the Study of Liver Diseases (AASLD) emphasized in its 2021 guidelines for the first time that nutritional management in cirrhosis can improve quality of life, delay disease progression, and treat complications (3). Although numerous studies have investigated nutritional assessment in cirrhotic patients (4–8), specific risk assessment and prognostic indicators remain lacking.

Traditional risk prediction methods rely primarily on regression analysis (9, 10). Given the complexity of factors influencing cirrhosis with esophagogastric varices, no definitive conclusions have been reached, necessitating the collection of extensive characteristics to determine patient bleeding risk. Extensive literature demonstrates that the eXtreme Gradient Boosting (XGBoost) algorithm serves as a powerful machine learning method that constructs strong learners by combining multiple weak learners (typically decision trees), thereby enhancing model prediction accuracy (11–13). This study employs XGBoost for model construction. However, as a complex “black box” model, XGBoost’s internal prediction process makes it difficult to intuitively understand each feature’s impact on prediction results. We utilized SHAP (SHapley Additive exPlanations) to interpret the XGBoost model characteristics and optimize model performance (14–16). SHAP clarifies the prediction generation mechanism of machine learning models such as XGBoost by quantifying each feature’s impact. By assigning a Shapley value to each feature, SHAP elucidates the positive or negative contribution of each feature to model prediction, thereby enhancing model interpretability. In disease research, the combination of XGBoost and SHAP has significantly improved prediction accuracy and interpretability. Assigning numerical values to each feature reveals the degree of deviation from prediction results, making model decision-making more transparent (13, 17–21).

Through machine learning, this study investigates whether nutritional status, different nutritional screening scales, and nutritional parameters affect bleeding and prognosis in cirrhotic patients with esophagogastric variceal bleeding (EGVB). The goal is to provide improved nutritional support treatment and prediction for such patients while constructing a nutrition-based prediction model to offer clinicians more effective predictions of bleeding risk and associated risk factors.

## 2 Materials and methods

### 2.1 Patients

We retrospectively analyzed data from 416 hospitalized cirrhotic patients admitted to the First Hospital of Shanxi Medical University between March 1, 2023, and March 31, 2025.

Inclusion criteria: (1) Definite diagnosis of cirrhosis by liver biopsy or imaging examination; (2) Nutritional scale evaluation and nutritional assessment performed during hospitalization; and (3) Patients with symptoms, ascites testing, abdominal ultrasound, head MRI, and abdominal CT scan + enhancement + CTV to clarify the presence of serious complications.

Exclusion criteria: (1) Combined heart, lung, or other multiple organ failure; (2) Inability to cooperate with treatment; (3) Significant lack of clinical data; and (4) Patients with liver failure.

A total of 135 patients with cirrhosis complicated by EGVB and 281 patients without EGVB were screened. This study was reviewed and approved by the Ethics Committee of the First Hospital of Shanxi Medical University.

Standardized EGVB definition (1). EGVB was defined as acute upper gastrointestinal bleeding with endoscopic confirmation of esophageal and/or gastric variceal bleeding (active bleeding or stigmata of recent hemorrhage), in line with the Baveno VII consensus. All EGVB cases were confirmed by emergency endoscopy within 24–48 h of admission; cases without endoscopic confirmation were excluded. A total of 135 patients with cirrhosis complicated by acute EGVB dc and 281 patients without acute EGVB were screened. This study was reviewed and approved by the Ethics Committee of the First Hospital of Shanxi Medical University.

### 2.2 Data collection

Clinical data were retrospectively collected, encompassing 52 variables including:

- Clinical characteristics: gender, age, etiology, disease duration, Child-Pugh score, MELD score, number of hospitalizations, presence of other serious complications during hospitalization (severe infection, ascites, hepatic encephalopathy, hepatocellular carcinoma).
- Laboratory indicators: complete blood count (white blood cells [WBC], neutrophils, lymphocytes, monocytes, platelets [PLT], hemoglobin [Hb]); routine biochemistry (alanine aminotransferase [ALT], aspartate aminotransferase [AST], serum albumin [Alb]); coagulation tests (prothrombin time [PT], prothrombin activity [PTA], international normalized ratio [INR]); other parameters (serum sodium [Na], serum potassium [K], blood urea nitrogen [BUN], serum creatinine [Scr]).
- Imaging-related parameters: liver-related conditions, portal vein diameter (PVD), etc.

### 2.3 Nutritional characteristics

All enrolled patients received nutritional assessment by two attending physicians with 15 and 25 years of clinical experience, respectively, on the day of admission. Since multiple malnutrition screening tools are widely applied in routine clinical work, we adopted eight commonly-used nutritional screening tools in this study. Table 1 summarizes the core screening contents of these eight tools, while their specific scoring rules are not displayed herein. The eight tools were Malnutrition Universal Screening Tool (MUST), Nutritional Risk Screening 2002 (NRS-2002), Royal Free Hospital-Nutritional Prioritizing Tool (RFH-NPT), Geriatric Nutritional Risk Index (GNRI), Nutritional Risk Index (NRI), Mini-Nutritional Assessment Short-Form (MNA-SF), Subjective Global Assessment (SGA) and Frailty Index (FI). All scoring criteria were implemented in accordance with established definitions and routine clinical practices.

**Table 1:** Summary of screening elements used in nutritional screening tools.

Assessment parameters	Weight	Alcohol or tube feeding	Age	BMI	Appetite loss	Disease severity	Edema	Food absorption	GI symptoms	Activity change	Neuropsychiatric issues	Stress response/acute illness	Weight change	TSF	Muscle wasting	Serum albumin
NRI	√															√
GNRI	√															√
MUST				√								√	√			
NRS-2002			√	√		√		√					√			
MNA-SF					√				√	√	√	√	√			
FI																
SGA							√	√	√	√	√	√	√	√	√	
RFH-NPT		√			√		√						√			

MUST, malnutrition universal screening tool; NRS-2002, nutritional risk screening 2002; MNA-SF, mini nutritional assessment short-form; FI, frailty index; SGA, subjective global assessment; RFH-NPT, royal free hospital-nutritional prioritizing tool; GNRI, geriatric nutritional risk index; NRI, nutritional risk index.

Other nutritional indicators: mid-upper arm circumference (MUAC), triceps skinfold thickness (TSF, mm), arm muscle circumference (AMC), grip strength (kg), and adjusted BMI. Baseline demographic data, laboratory examination indicators, and nutritional evaluation parameters were collected.

### 2.4 Statistical analysis

#### 2.4.1 Data preprocessing

Dataset was split into training and test sets via stratified sampling at an 8:2 ratio according to the outcome variable (EGVB). A fixed random seed was set to ensure reproducibility. To address the class imbalance in the training set, scale_pos_weight (the ratio of the number of negative samples to positive samples) was calculated and used as the class weight parameter for the XGBoost model to mitigate the bias induced by imbalanced class distribution during model training.

#### 2.4.2 Construction of XGBoost prediction model and feature selection

First, a modeling pipeline was established using Scikit-learn. The pipeline consisted of a StandardScaler normalization module and an XGBoost binary classifier. A full-feature XGBoost model was then built with all candidate features. Permutation Importance was subsequently adopted for feature selection. Using AUC as the evaluation metric, permutation importance of each feature was calculated for 30 repeated runs. The top 10 features ranked by importance were selected to construct the optimal feature subset model. Hyperparameters of this subset model were kept identical to those of the full-feature model to enable direct comparison between the two models.

Model performance was assessed by AUC, accuracy, precision, recall, F1-score, Brier score, specificity, positive predictive value (PPV), and negative predictive value (NPV). ROC curves, calibration curves, and decision curve analysis (DCA) were plotted to comprehensively evaluate the model’s discrimination, calibration, and clinical utility.

The model was assessed with multiple metrics including AUC, accuracy, precision, recall, F1-score and Brier score. ROC curves, calibration curves, and decision curve analysis (DCA) were plotted to comprehensively evaluate the model’s discriminative ability, calibration performance and clinical utility.

#### 2.4.3 SHAP analysis

SHAP theory originates from cooperative game theory and provides a rigorous, highly interpretable tool to quantify the specific effect and relative importance of each feature on model predictions. SHAP (SHapley Additive exPlanations) was applied to interpret the optimal feature-subset XGBoost model. The SHAP summary plot illustrates the direction and magnitude of feature contributions to predictions. The SHAP importance bar chart ranks features according to the mean absolute SHAP values. The waterfall plot intuitively demonstrates the positive and negative contributions of each feature to the prediction for an individual sample. The SHAP heatmap visualizes the distribution of SHAP values across all samples and features. The SHAP decision plot presents the decision path from the base value to the final predicted probability for multiple samples.

### 2.5 Statistical analysis

All statistical analyses were performed using Python 3.9 (libraries: scikit-learn, XGBoost, imbalanced-learn, SHAP). Two-tailed tests were adopted, and *p* < 0.05 was considered statistically significant. Continuous variables were described as mean ± standard deviation (Mean ± SD) or median (interquartile range) [M (P25, P75)]. Categorical variables were reported as frequency (percentage) [n (%)]. Univariate logistic regression was used to assess the association between each nutritional assessment tool and EGVB occurrence. Receiver Operating Characteristic (ROC) curves were used to evaluate the diagnostic performance of each nutritional assessment tool, and the corresponding Area Under the Curve (AUC) with 95% confidence interval (95% CI) was calculated (Figure 1).

**Figure 1:**
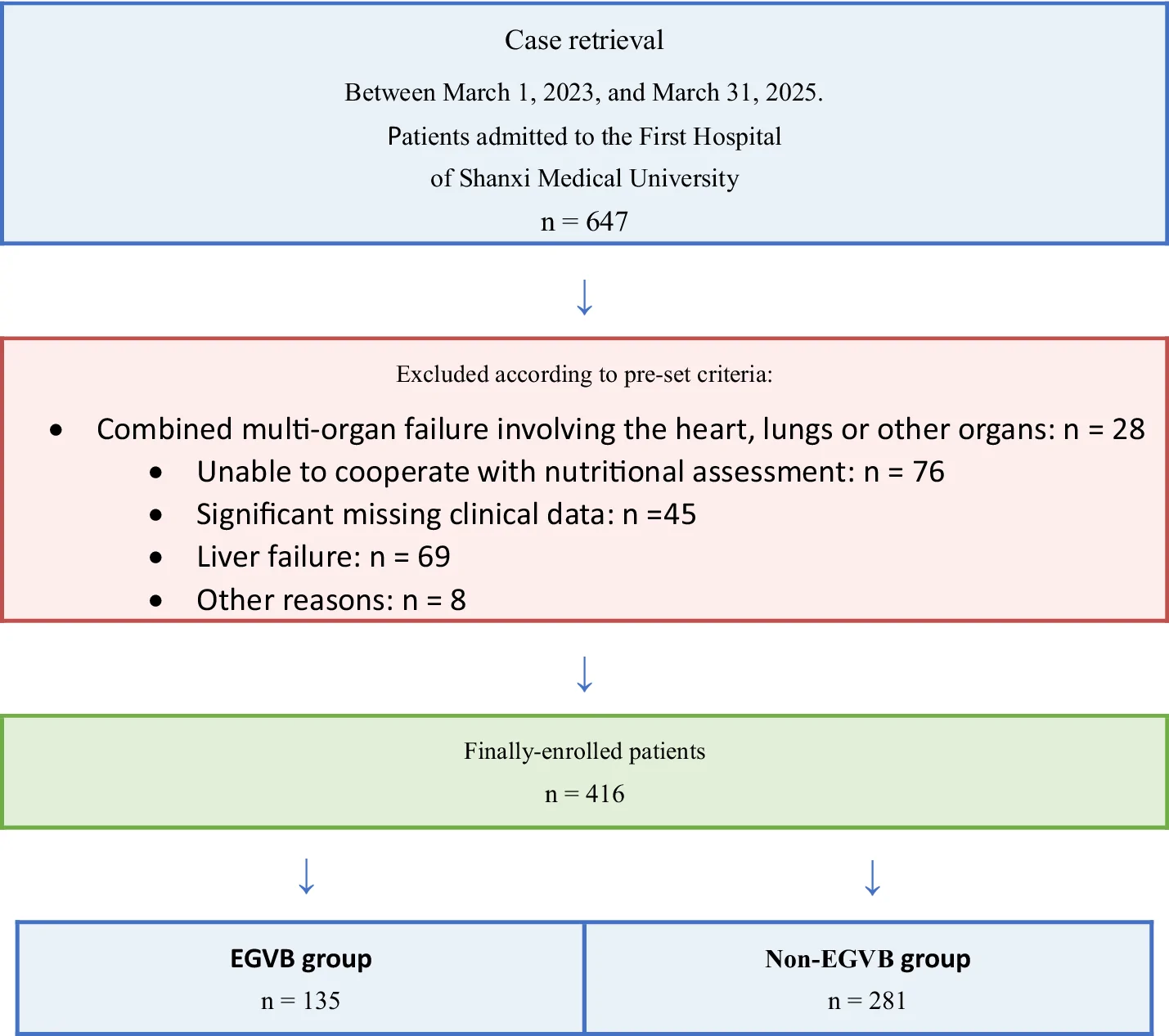
Flow diagram of patient selection.

### 2.6 Ethical approval

This retrospective study was approved by Ethics Committee of First Hospital of Shanxi Medical University (No. KYLL-2026-191). The requirement for informed consent was waived because of the retrospective design. All data were analyzed anonymously.

## 3 Results

### 3.1 Baseline characteristics of the two groups

A total of 416 cirrhotic patients were included in this study: 135 (32.5%) in the esophagogastric variceal bleeding (EGVB) group and 281 (67.5%) in the non-EGVB group. The mean age was 56.65 ± 11.86 years in the EGVB group vs. 59.04 ± 11.53 years in the non-EGVB group. Female patients accounted for 63% versus 65.1%, respectively. The primary etiology of cirrhosis was viral infection (44.4% vs. 38%). Comparisons of baseline demographic characteristics, clinical manifestations, and laboratory indicators between the two groups are shown in Table 2.

**Table 2:** Comparison of clinical characteristics between EGVB and non-EGVB groups.

Characteristics	EGVB group (*n* = 135)	Non-EGVB group (*n* = 281)	*P* value
Gender (Female, %)	63%	65.10%	0.664
Age (years)	56.65 ± 11.86	59.04 ± 11.53	0.818
Length of hospital stay (days)	10.89 ± 0.525	11.11 ± 0.36	0.730
Number of hospitalizations	1.96 ± 0.155	2.15 ± 0.129	
First admission/Readmission for cirrhosis, *n* (%)	40%/60%	49.8%/50.1%	0.074
3-month mortality (Yes, *n*, %)	2.90%	4.20%	0.598
Hepatic encephalopathy (No, *n*, %)	94%	89.30%	0.146
Hepatocellular carcinoma (No, *n*, %)	85.10%	81.80%	0.487
Infection (No, *n*, %)	91.80%	80.40%	0.002**
Ascites (No, *n*, %)	32.50%	28.85	0.067
Etiology, *n* (%)			0.070
Viral hepatitis, *n* (%)	44.40%	38%	
Alcoholic liver disease, *n* (%)	12.50%	23.10%	
Autoimmune liver disease, *n* (%)	16.20%	17%	
Others, *n* (%)	26.60%	21.70%	
NRI	91.76 (84.84–99.47)	91.76 (86.15–96.85)	0.976
GNRI	89.92 (84.94–94.86)	90.83 (82.01–97.84)	0.850
MUST score (0/1/2)	34.8%/4.4%/60.7%	54.8%/14.9%/30.2%	<0.001 **
FI	24.4%/29.6%/45.9%	46.2%/23.1/30.2%	<0.001 ***
NRS-2002 (high-risk, %)	36.20%	51.90%	0.003**
RFH-NPT (Low/Moderate/High risk), *n* (%)	14.8%/17.7%/67.4%	16.7%/29.1%/54%	0.026 *
MNA-SF (Low/Moderate/High risk), *n* (%)	42.9%/45.9/11.1%	41.2%/44.4%/14.2%	0.503
SGA (A/B/C), *n* (%)	73.3%/22.2%/4.4	59.2%/39.2%/3.9%	0.029*
Mid-upper arm circumference (cm)	27.0 (24.0–29.75)	26.0 (23.0–29.0)	0.155
Triceps skinfold thickness (mm)	14 (11.0–20.0)	14 (10.0–22.0)	0.986
Mid-arm muscle circumference (cm)	21.86 (18.84–24.6)	20.86 (18.09–24.3)	0.081
Adjust BMI (kg/m^2^)	21.66 ± 0.239	22.136 ± 0.339	0.222
Child-Pugh grade (A/B/C)	51.8%/22.9%/25.1%	22%/45.9%/32%	0.343
MELD score	8.72 ± 6.38	8.72 ± 8.23	0.641
Portal vein diameter (cm)	1.369 ± 0.021	1.375 ± 0.031	
White blood cells (×10^9^/L)	3.01 (2.5–3.51)	3.43 (2.84–4.06)	<0.001***
Red blood cell count (×10^12^/L)	3.3 (2.1–5.3)	4.4 (2.8–5.95)	0.002**
Platelets (×10^9^/L)	91.0 (56.0–136.0)	67 (49–113)	0.005**
Neutrophils (×10^9^/L)	1.9 (1.2–3.35)	2.65 (1.56–3.76)	0.003**
Lymphocytes (×10^9^/L)	0.77 (0.48–1.07)	0.99 (0.65–1.46)	<0.001***
Monocytes (×10^9^/L)	0.29 (0.2–0.5)	0.35 (0.24–0.51)	0.054
Hemoglobin (g/L)	91.34 ± 25.66	109.32 ± 26.93	<0.001***
Prothrombin time (s)	15.82 ± 3.24	15.87 ± 3.50	0.906
Prothrombin activity (%)	61.3 (51.0–71.3)	62.14 (49–72.5)	0.970
INR	1.33 (1.17–1.47)	1.33 (1.17–1.52)	0.721
ALT (U/L)	23.0 (14.0–39.5)	26.0 (15.0–50.0)	0.035
AST (U/L)	32 (25, 49.5)	42 (28, 80)	<0.001***
Albumin (g/L)	31.70 ± 4.95	31.54 ± 5.24	0.717
Creatinine (μmol/L)	64.4 (56.4–77.25)	60.9 (51.0–73.0)	0.009**
Blood Urea (mmol/L)	5.39 (3.84–7.06)	5.13 (3.79–6.7)	0.566
Sodium (mmol/L)	139.0 (136.0–141.0)	140.0 (137.0–142.0)	0.179
Potassium (mmol/L)	3.809 ± 0.031	3.876 ± 0.037	0.139

“First admission” was defined as the index hospitalization for cirrhosis or any of its complications; “readmission” was defined as any subsequent hospitalization for cirrhosis or its complications. MUAC, mid-upper arm circumference; TSF, triceps skinfold thickness; AMC, arm muscle circumference; BMI, body mass index; WBC, white blood cells; PLT, platelets; Hb, hemoglobin; ALT, alanine aminotransferase; AST, aspartate aminotransferase; Alb, albumin; PT, prothrombin time; PTA, prothrombin activity; INR, international normalized ratio; BUN, blood urea nitrogen; Scr, serum creatinine; Na, sodium; K, potassium; * *p* < 0.05, ** *p* < 0.01, *** *p* < 0.001.

### 3.2 Comparison of predictive performance among different nutritional screening tools

Given content overlap across the eight nutritional assessment tools, univariate logistic regression was performed for each nutritional-screening indicator against EGVB to mitigate multicollinearity interference for subsequent XGBoost model construction. ROC curves were plotted based on the predicted probability of each model.

As shown in Table 3 and Figure 2, MUST (OR 1.444, 95% CI: 1.222–1.705; *p* < 0.001), FI (OR 1.538, 95% CI: 1.173–2.017; *p* = 0.002), NRS-2002 (OR 1.688, 95% CI: 1.058–2.693; *p* = 0.028) and SGA (OR 0.646, 95% CI: 0.422–0.991; *p* = 0.045) were significantly associated with the occurrence of EGVB in cirrhosis. MUST exhibited the best discriminative performance with an AUC of 0.698, followed by FI (AUC = 0.683), NRS-2002 (AUC = 0.634), and RFH-NPT (AUC = 0.621). The AUC values of other nutritional-screening indicators were all below 0.6.

**Table 3:** Univariate logistic regression analysis of different nutritional assessment scales.

Feature	OR	95%CI	*P* value	*β*	AUC	Brier
MUST	1.444	1.222, 1.705	<0.001***	0.37	**0.698**	**0.190**
FI	1.538	1.173, 2.017	0.002 **	0.43	0.683	0.201
NRS-2002	1.688	1.058, 2.693	0.028**	0.52	0.634	0.208
RFH-NPT	1.157	0.955, 1.401	0.136	0.15	0.621	0.212
SGA	0.646	0.422, 0.991	0.045*	−0.44	0.558	0.216
GNRI	1.001	0.998, 1.003	0.674	0.00	0.515	0.219
NRI	1.002	0.981, 1.023	0.857	0.00	0.483	0.218
MNA-SF	0.820	0.584, 1.151	0.251	−0.20	0.442	0.221

OR, odds ratio; CI, confidence interval; * *p* < 0.05, ** *p* < 0.01, *** *p* < 0.001. Bold font indicates the optimal value for the corresponding indicator.

**Figure 2:**
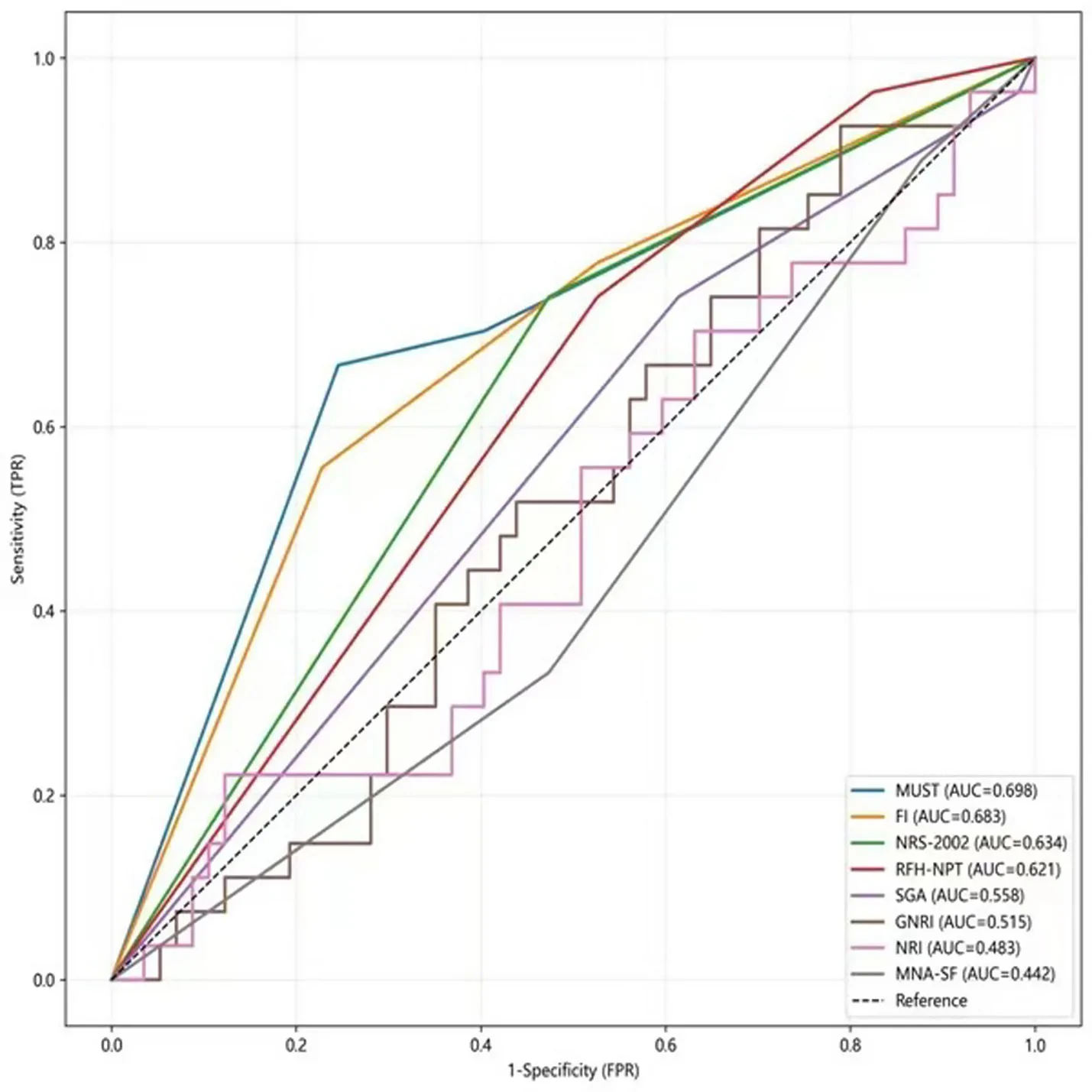
ROC curves of eight nutritional screening tools for predicting EGVB.

Considering its stronger association with EGVB, MUST was incorporated into the XGBoost model for further analyses.

### 3.3 Model construction

An XGBoost model was built incorporating all variables, and feature screening was performed to develop a simplified model.

As shown in Table 4, a gradient-boosting classifier model was constructed using all 36 clinical indicators. For the Baseline XGBoost model on the test set, the AUC was 0.839, accuracy 79.5%, recall 59.3%, precision 72.7%, F1-score 65.3%, and Brier score 0.145. The optimal XGBoost model selected the top-ranked 10 indicators to build the simplified model: hemoglobin, MUST score, AST, serum creatinine, adjusted BMI, ALT, neutrophils, potassium (K+), monocyte count, and mid-arm muscle circumference. On the test set, this simplified model yielded an AUC of 0.860, accuracy 84.3%, recall 66.7%, precision 81.8%, F1-score 73.5%, and Brier score of 0.122.

**Table 4:** Performance of the full-indicator model and the optimal model.

Model	AUC	Accuracy	Recall	Precision	F1-score	Brier
Baseline XGBoost	0.839	0.795	0.593	0.727	0.653	0.145
Initialized XGBoost	0.860	0.843	0.667	0.818	0.735	0.122

The AUC difference between the Baseline XGBoost model and the optimal model was 0.021. Using only ten predictors, the optimal model achieved better performance than the full-variable model and was more cost-effective.

Figure 3 shows the ROC curve (Figure 3A), calibration curve (Figure 3B), and clinical decision curve (Figure 3C) of the optimal model. The XGBoost model constructed in this study achieved an AUC of 0.860, indicating favorable discriminative performance. The calibration curve (Figure 3B) revealed good overall consistency between the predicted probabilities and the actual observed event probabilities of the model. The decision-curve analysis (DCA, Figure 3C) demonstrated that within a wide range of threshold probabilities, risk-stratified intervention guided by this model yielded greater net benefit than the strategies of universal intervention or no-intervention. This model has potential value for clinical application.

**Figure 3:**
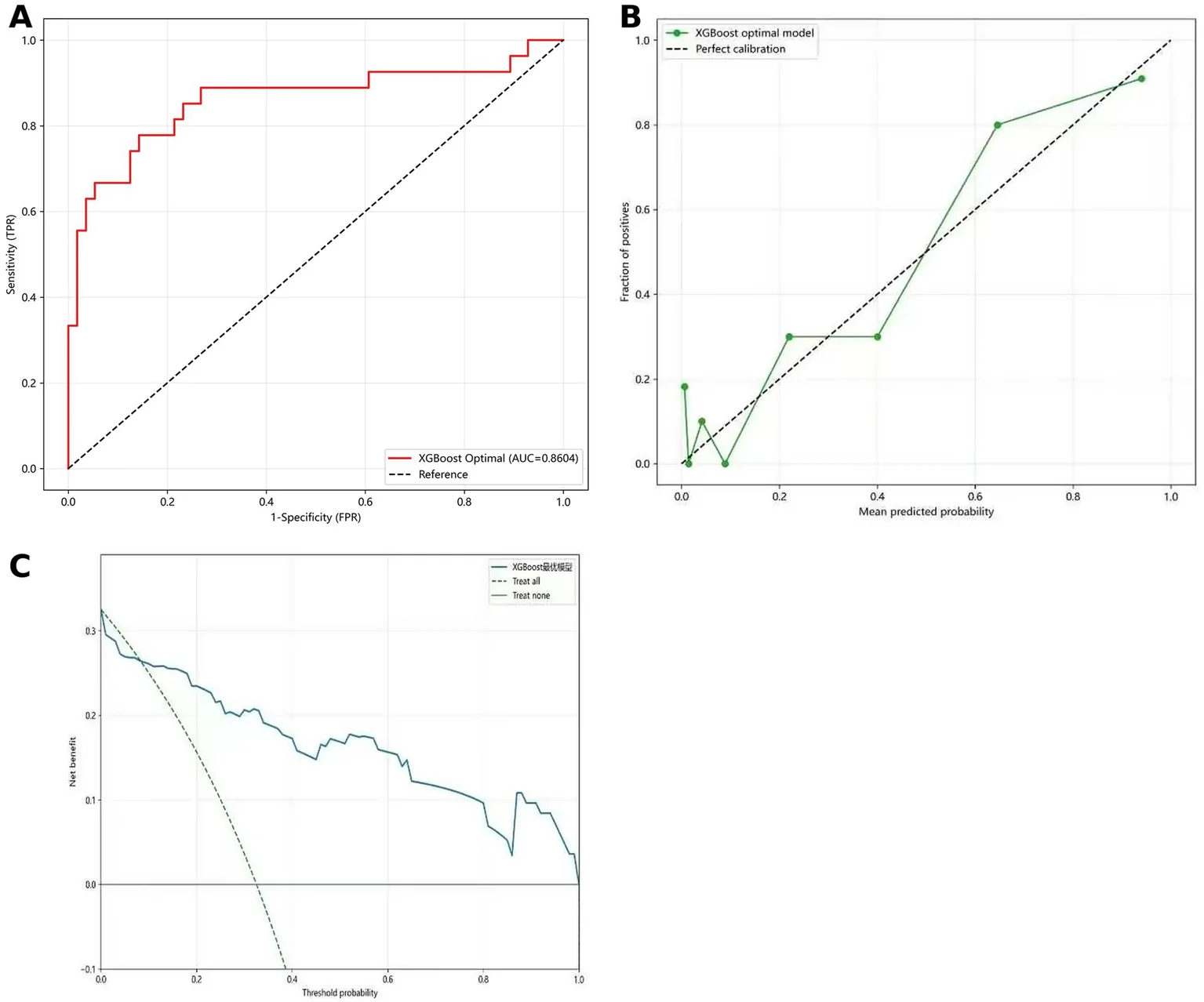
Comprehensive evaluation of the predictive performance of the XGBoost model using ROC, calibration and decision curves. **(A)** ROC curve: horizontal axis, 1-specificity; vertical axis, sensitivity. AUC closer to 1 indicates better discriminative performance. **(B)** Calibration curve: horizontal axis, model-predicted probability; vertical axis, observed probability. The 45° dashed line is the ideal calibration; greater overlap between the solid and dashed lines means superior calibration. **(C)** Decision curve: horizontal axis, threshold probability; vertical axis, net benefit. “Treat All” = universal intervention; “Treat None” = no intervention. A model curve above both reference lines indicates clinical net benefit.

### 3.4 SHAP interpretation

Optimal XGBoost model and SHAP analysis (Figure 4):

**Figure 4:**
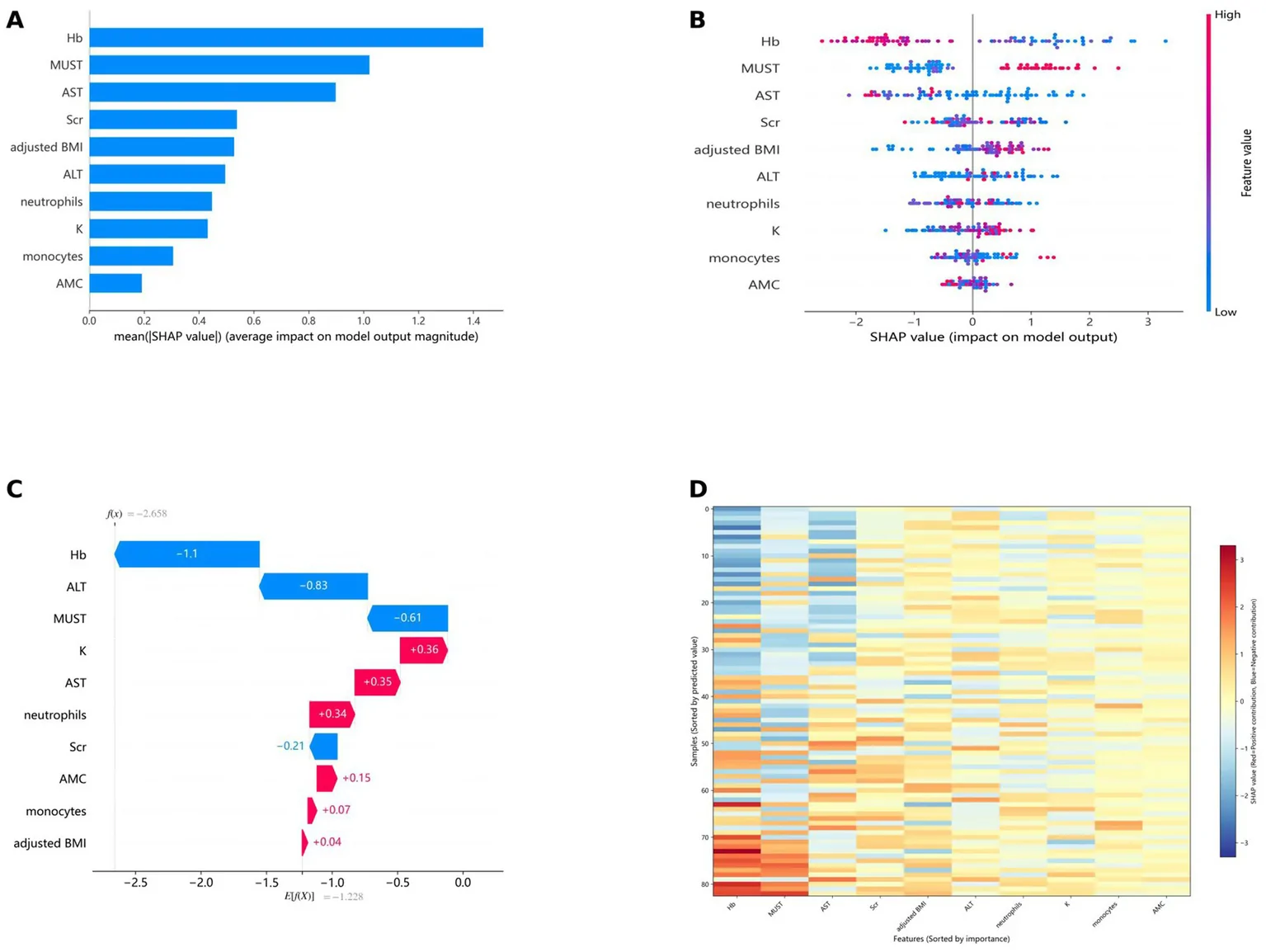
SHAP-based interpretability analysis of the XGBoost model for EGVB risk prediction. **(A)** Variable importance ranking of the XGBoost model. **(B)** Summary plot of feature impact direction and magnitude on EGVB risk. **(C)** Individual sample explanation plot for predicted EGVB risk. **(D)** Heatmap of feature contribution patterns across individual samples.

The SHAP summary plot (Figure 4A) showed that hemoglobin (Hb) and MUST score were the two most important predictors for EGVB, followed by AST, serum creatinine, adjusted BMI, ALT, neutrophils, K^+^, monocyte count, and mid-arm muscle circumference (AMC). In the predictive model, MUST score, adjusted BMI and AMC highlighted the critical role of nutritional status in EGVB prediction.

The SHAP dependence summary plot (Figure 4B) shows the effect direction of each variable for EGVB risk prediction. MUST score, Scr, adjusted BMI, ALT and serum potassium acted as positive predictors, whereas Hb, AST, neutrophil count, monocyte count and AMC were negative predictors.

The SHAP waterfall plot (Figure 4C) interprets the XGBoost-predicted EGVB risk for a single patient. The baseline value E[f(X)] = −1.228 corresponds to the model’s mean prediction over the entire cohort. For this case, hemoglobin (SHAP value = −1.10), MUST score (−0.83), ALT (−0.61), and serum creatinine (−0.21) constituted the major negatively contributing features, with hemoglobin acting as the leading factor reducing EGVB risk. Potassium ions (+0.36), AST (+0.35), neutrophil count (+0.34), AMC (+0.15), monocyte count (+0.07), and adjusted BMI (+0.04) were positively contributing features. The final predicted value f(x) = −2.658 was substantially lower than the baseline, suggesting a low probability of EGVB in this patient.

The SHAP heatmap (Figure 4D) further visualizes the feature contribution matrix at the individual sample level. MUST and Hb showed consistent directional effects in the majority of samples, while other features demonstrated more variable impacts according to individual patient profiles.

## 4 Discussion

We developed an XGBoost-SHAP integrated prediction model for EGVB in patients with liver cirrhosis. The model achieved high outcomes in terms of AUC, accuracy, recall, precision, as well as a lower Brier score, exhibiting good discrimination and calibration. The optimal machine learning model incorporating hemoglobin, MUST score, AST, Scr, adjusted BMI and other clinical parameters achieved excellent predictive accuracy (AUC = 0.860). SHAP analysis revealed that hemoglobin and MUST score were the most influential predictors. In addition to the ranking of feature importance, the direction of certain feature contributions warrants clarification. AST contributed negatively to EGVB risk in the model, a direction consistent with the lower median AST observed in the EGVB group (Table 2). This likely reflects differences in the baseline characteristics between the two hospitalized cirrhotic populations—for instance, the higher proportion of patients with infection, ascites, or hepatic encephalopathy in the non-EGVB group—rather than a protective effect of AST itself. As with all SHAP-derived directions, this association describes the model’s behavior within this cohort and should not be interpreted as causal. These findings establish a robust, interpretable framework for EGVB risk stratification, filling a critical gap between nutritional assessment and bleeding risk prediction in patients with cirrhosis. Previous model construction (9, 10) primarily relied on traditional risk prediction methods (such as logistic regression and Cox proportional hazards models), which present obvious limitations. Machine learning has revolutionized disease prediction paradigms, particularly in identifying high-risk patients—an especially important application in clinical environments where endoscopy is restricted according to non-invasive screening criteria. Previous studies have attempted to apply machine learning algorithms to predict cirrhosis-related complications, including the research by Agarwal et al. (22) and Wang et al. (23). However, these studies exclusively adopted endoscopic parameters and endoscopic images as model input variables, and few machine learning prediction models based on clinical nutritional indicators have been reported. Compared with previous studies, the present study screened and compared eight commonly used clinical nutritional assessment and screening tools. By constructing an integrated XGBoost-SHAP model, this study overcomes the limitations of conventional prediction methods and establishes a novel EGVB prediction model combining nutritional indicators (MUST score, adjusted BMI, and mid-arm muscle circumference) with multiple non-invasive clinical parameters. Notably, the predictive performance of the present model (AUC = 0.860) is comparable to that of the endoscopic parameter-based XGBoost model reported by Agarwal et al. (22) (AUC = 0.857). More importantly, relying entirely on non-invasive indicators, our model avoids the limitations of endoscopic examination, including low patient acceptance and potential procedural risks. Therefore, it is more suitable for routine EGVB screening and grassroots clinical promotion, possessing promising clinical transformation value.

Notably, in addition to achieving non-invasive prediction, the predictive factors screened by this model include three nutritional indicators, namely MUST score, adjusted BMI, and AMC, alongside conventional clinical and laboratory parameters. Further analysis revealed that these three indicators are mutually complementary, and their combined assessment enables a more comprehensive evaluation of patients’ nutritional status. Specifically, although the MUST score serves as a comprehensive nutritional risk screening tool, its BMI component is susceptible to interference from ascites. By contrast, adjusted BMI and AMC precisely compensate for this limitation by reflecting body composition and muscle reserve, respectively (24). Our analytical results demonstrated that cirrhotic patients with high MUST scores and EGVB did not present with traditional low-body-weight malnutrition. Instead, they exhibited relative body composition imbalance, characterized by elevated adjusted BMI and decreased AMC within the cohort. This finding indicates that high nutritional risk combined with fat excess and muscle depletion collectively contributes to the occurrence of EGVB.

Accumulating evidence from clinical cohort and animal studies has suggested that malnutrition, Sarcopenia, and excess fat independently contribute to the development and progression of liver cirrhosis, portal hypertension, and esophagogastric variceal bleeding (EGVB).

In terms of malnutrition, a study involving 151 patients with acute EGVB further found that 97.4% of the participants had malnutrition, and the degree of nutritional risk was positively correlated with PT, INR, D-dimer, and IL-6 levels, indicating that malnutrition may directly aggravate bleeding risk through inflammatory activation and coagulation imbalance (25). Animal experiments (26, 27) have revealed the molecular mechanisms underlying this association. A low-protein diet induces hepatic steatosis, inflammation, and fibrosis by up-regulating *de novo* lipogenesis, enhancing oxidative stress, and inhibiting mitochondrial biogenesis via the down-regulation of PGC1α, TIM23, and TFAM. In addition, starvation models have confirmed that a 25% reduction in body weight can trigger elevated AST/ALT levels, hepatic glycogen depletion, and enhanced autophagy, leading to direct hepatocellular injury (28). Furthermore, a review by Albillos A et al. (29) suggested that systemic inflammation contributes to hemodynamic disturbances in cirrhosis and promotes the progression of portal hypertension (30, 31), which is the predominant pathogenic mechanism responsible for the occurrence and progression of EGVB (32). Collectively, malnutrition induces liver injury and activates systemic inflammatory responses, thereby disrupting hemodynamic stability, facilitating the development of portal hypertension, and ultimately participating in the pathogenesis of EGVB.

With regard to sarcopenia, an international multicenter cohort study involving 1,624 cirrhotic patients recruited from Canada, South Korea, India, and Netherlands demonstrated that the incidence of variceal bleeding was significantly higher in the sarcopenia group than in the non-sarcopenia group (30% vs. 23%, *p* = 0.003) (33). A multicenter cohort study based on propensity score matching further validated that sarcopenia serves as an independent prognostic factor for recurrent variceal bleeding after endoscopic treatment (34).

In terms of fat excess, clinical-radiomic studies of decompensated cirrhosis have shown that patients with sarcopenic obesity carry a 5- to 6-fold higher risk of upper gastrointestinal bleeding than non-sarcopenic obese patients, indicating that the composite phenotype of concurrent fat excess and muscle depletion is closely associated with bleeding events (35). The association between this phenotype and portal hypertension has been further investigated in a Chinese multicenter cohort. Specifically, CT-measured intramuscular fat deposition, rather than muscle mass itself, is significantly correlated with the severity of portal hypertension. Patients with intramuscular fat deposition exhibited a markedly higher HVPG than those without fat deposition (21.57 ± 8.20 vs. 16.13 ± 6.89 mmHg), and intramuscular fat deposition was more prevalent in patients with HVPG ≥ 10 mmHg (36). Mechanistically, hepatic steatosis—the hepatic manifestation of systemic fat excess—drives the progression of portal hypertension mainly through two pathways. First, it induces hepatic sinusoidal endothelial dysfunction and remodeling, characterized by increased levels of vasoconstrictive factors including endothelin-1 (ET-1), angiotensin II, and thromboxane A2, as well as impaired endothelium-dependent vasodilation (37, 38). Second, fat excess triggers inflammatory responses. Lipid accumulation promotes excessive generation of reactive oxygen species (ROS) and oxidative stress, thereby causing steatohepatitis, hepatocellular injury, and hepatocellular ballooning. These pathological changes ultimately activate hepatic stellate cells, accelerate hepatic fibrosis, and further aggravate portal hypertension (39).

Notably, the three indicators identified in our study—the MUST score, AMC, and adjusted BMI— correspond conceptually to the three lines of evidence discussed above: nutritional risk assessed by MUST is closely related to malnutrition, reduced AMC is associated with sarcopenia, and adjusted BMI mirrors excess adiposity. Although each captures only a partial dimension of its corresponding phenotype, their combined assessment may more comprehensively reflect patients’ nutritional and metabolic status, showing a closer association with EGVB than any single measure. Moreover, unlike the full diagnostic criteria, these three indicators are inexpensive, non-invasive, and readily available in routine practice, underscoring their practical value for EGVB risk stratification.

However, several limitations of this study should be acknowledged. First, the retrospective design introduces potential selection bias and unmeasured confounding factors such as genetic factors and drug interactions, which may affect EGVB risk. Further prospective studies are warranted to control for these confounding variables. Second, endoscopic confirmation of esophagogastric varices was not available for the non-EGVB group, as performing gastroscopy in patients without bleeding was not justifiable under our ethics approval given its procedural risks. Third, emergency interventions such as blood transfusion and fluid resuscitation before admission sampling may have partially influenced baseline laboratory values. Finally, inter-rater variability in AMC measurements was not fully assessed in this study, and dynamic post-intervention follow-up data remain lacking. Future multicenter prospective cohort studies are warranted to explore the prognostic value of dynamic changes in AMC. Moreover, further research should validate the clinical utility of the combined model of adjusted BMI, AMC and MUST in guiding targeted nutritional interventions—such as branched-chain amino acid supplementation and nocturnal feeding—to improve clinical prognosis.

## 5 Conclusion

In summary, the nutritional indicator-based prediction model developed in this study exhibits favorable accuracy and clinical feasibility for predicting EGVB in cirrhotic patients. Its predictive performance is superior to that of single traditional nutritional screening tools. This explainable and noninvasive machine learning method provides a reliable tool for clinical risk stratification and individualized intervention, which is expected to improve clinical outcomes in this high-risk population. This study confirms that three nutritional indicators, namely the MUST score, AMC and adjusted BMI, are closely associated with the risk of EGVB in cirrhotic patients. Combined nutritional assessment using multiple indicators facilitates clinical risk stratification of EGVB.

## Data Availability

The datasets analyzed for this study are not publicly available due to patient privacy and confidentiality concerns. The data were obtained from medical records at First Hospital of Shanxi Medical University and contain potentially identifiable clinical information. As such, access to the data is restricted. Requests for access to the de-identified dataset may be directed to the corresponding author (HH, huanghuifang@sydyy.com) and will be considered on a case-by-case basis, subject to institutional and ethical approval.
